# CRISPR Interference Directs Strand Specific Spacer Acquisition

**DOI:** 10.1371/journal.pone.0035888

**Published:** 2012-04-27

**Authors:** Daan C. Swarts, Cas Mosterd, Mark W. J. van Passel, Stan J. J. Brouns

**Affiliations:** 1 Laboratory of Microbiology, Department of Agrotechnology and Food Sciences, Wageningen University, Wageningen, The Netherlands; 2 Systems and Synthetic Biology, Department of Agrotechnology and Food Sciences, Wageningen University, Wageningen, The Netherlands; St. Petersburg Pasteur Institute, Russian Federation

## Abstract

**Background:**

CRISPR/Cas is a widespread adaptive immune system in prokaryotes. This system integrates short stretches of DNA derived from invading nucleic acids into genomic CRISPR loci, which function as memory of previously encountered invaders. In *Escherichia coli*, transcripts of these loci are cleaved into small RNAs and utilized by the Cascade complex to bind invader DNA, which is then likely degraded by Cas3 during CRISPR interference.

**Results:**

We describe how a CRISPR-activated *E. coli* K12 is cured from a high copy number plasmid under non-selective conditions in a CRISPR-mediated way. Cured clones integrated at least one up to five anti-plasmid spacers in genomic CRISPR loci. New spacers are integrated directly downstream of the leader sequence. The spacers are non-randomly selected to target protospacers with an AAG protospacer adjacent motif, which is located directly upstream of the protospacer. A co-occurrence of PAM deviations and CRISPR repeat mutations was observed, indicating that one nucleotide from the PAM is incorporated as the last nucleotide of the repeat during integration of a new spacer. When multiple spacers were integrated in a single clone, all spacer targeted the same strand of the plasmid, implying that CRISPR interference caused by the first integrated spacer directs subsequent spacer acquisition events in a strand specific manner.

**Conclusions:**

The *E. coli* Type I-E CRISPR/Cas system provides resistance against bacteriophage infection, but also enables removal of residing plasmids. We established that there is a positive feedback loop between active spacers in a cluster – in our case the first acquired spacer - and spacers acquired thereafter, possibly through the use of specific DNA degradation products of the CRISPR interference machinery by the CRISPR adaptation machinery. This loop enables a rapid expansion of the spacer repertoire against an actively present DNA element that is already targeted, amplifying the CRISPR interference effect.

## Introduction

Prokaryotes have evolved an adaptive immune system called CRISPR/Cas (clustered regularly interspaced short palindromic repeats and CRISPR associated protein) that enables them to counter invasions from viruses and plasmids (reviewed by [1], [2], [3], [4]). This immune system contains genomic CRISPR loci in which genetic material from invaders is incorporated. Memorized invaders can be recognized by expressing incorporated genetic material as small RNA molecules, which can guide Cas protein complexes to invader nucleic acid sequences.

The *E. coli* K12 genome encodes only a Type I-E CRISPR/Cas system [5], [6]. This system is capable of providing resistance to bacteriophage infection, prophage induction and plasmid transformation [7], [8], [9]. Comparative genomics has shown that the *E. coli* K12 genome contains two CRISPR loci with type 2 repeats and a variable spacer content (CRISPR locus 2.1 (12 spacers) and 2.3 (6 spacers)), suggesting that both loci are active [5], [10]. CRISPR locus 2.1 is located directly downstream of a Cas gene operon, while locus 2.3 does not have any *cas* genes encoded in its proximity. Both CRISPR loci have a conserved AT-rich leader sequence that acts as a promoter [11] and consist of 29 nucleotide palindromic repeats that are separated from each other by 32 or 33 nucleotide guide sequences called spacers. CRISPR transcripts are cleaved into mature CRISPR RNAs (crRNA) and these remain bound by the ribonucleoprotein complex Cascade (Cas-complex for antiviral defence, in Type I-E consisting of proteins encoded by *cas* genes *cse1*, *cse2*, *cas7*, *cas5* and *cas6e*) to guide the interference machinery to target DNA sequences (*i.e.* protospacers) [12]. In addition to Cascade, resistance requires the nuclease and helicase Cas3 [7], [13], [14]. Cas3 is recruited to the target DNA by the Cascade protein Cse1, after which Cas3 nicks the target DNA and further degrades the target DNA by ATP-dependent helicase and ssDNA nuclease activities [15].

Transcription of the Type I-E Cascade-*cas1*-*cas2* operon, and to some extent the CRISPR array, is repressed in this strain by the global transcriptional repressor H-NS (heat-stable nucleoid-structuring protein [11], [16], [17]. In the *hns* knock-out strain of *E. coli* K12 repression of the Cas genes is at least partially relieved [17], resulting in an activated CRISPR/Cas phenotype. Although the expression and interference stages of CRISPR immunity have been studied in *E. coli*, the process of acquiring spacers to modify the viral and plasmid specificity of the immune system has not yet been described.

The *Streptococcus thermophilus* Type II system integrates new spacers against bacteriophages [1], [18] and plasmids [19], and thereby acquires resistance to these bacteriophages (BIM: bacteriophage insensitive mutant) or cures itself from the corresponding plasmids (PIM: plasmid interfering mutant). The Type II specific Cas protein Csn2 [6], a calcium-dependent dsDNA binding protein [20], was reported to be essential during the spacer integration process in *S. thermophilus* [1]. In *E. coli*, Cas1 and Cas2 are not required during CRISPR expression or interference [7]. Their strict conservation with CRISPR loci suggests involvement in CRISPR adaptation [21].

Here we describe that *E. coli* K12 Δ*hns* is cured from a high copy number plasmid by integrating new spacers into two CRISPR loci. Based on our observations we propose that active spacers in a cluster are used to expand the range of new spacers against the same target in a strand specific manner.

## Results and Discussion

### Spacer Integration Results in Plasmid Curing and Plasmid Interference

**Figure 1:**
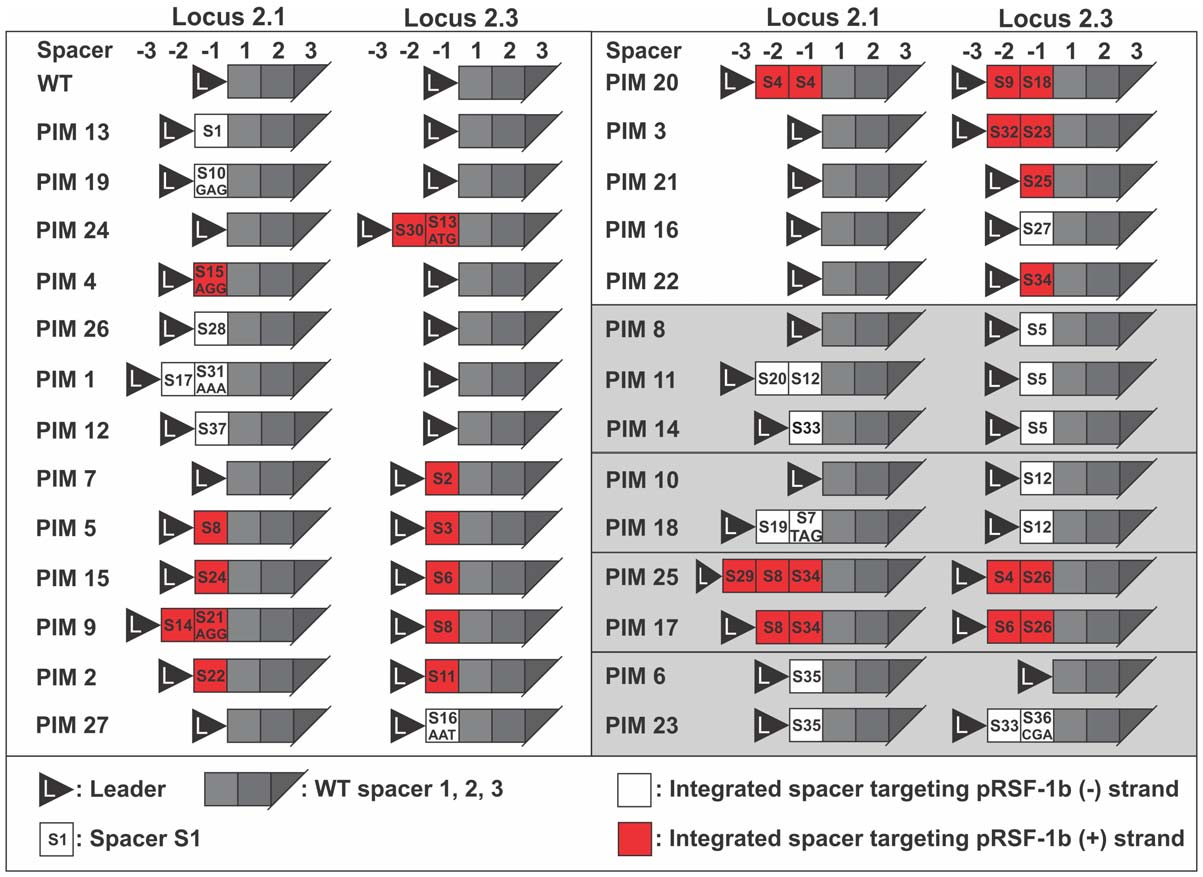
Graphical representation of spacers integrated in the various PIMs. Both CRISPR locus 2.1 and 2.3 of each PIM are displayed. The newly acquired spacer positions (−3, −2, −1) and original spacer positions (1, 2, 3) correspond to the order of spacers downstream from the leader sequence (displayed as black triangle). White and red spacer boxes indicate that the corresponding protospacer is located on the – or + strand of the plasmid, respectively. PIMs clustered in grey boxes possibly share a common ancestor. Spacers have an AAG PAM unless indicated otherwise. Additional information on spacers is given in Table S1.

**Figure 2:**
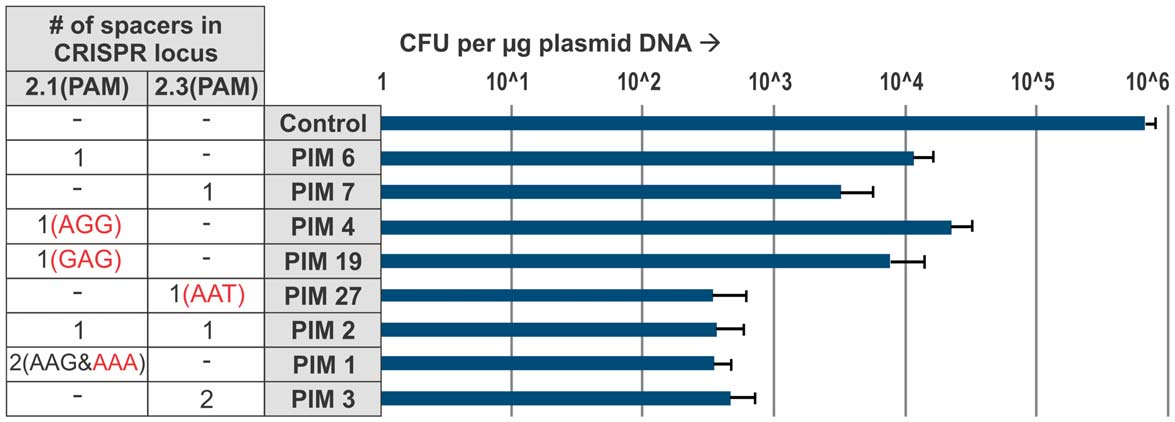
Effect of integrated spacers on retransformation efficiency. Transformation efficiencies of various PIMs and the control (Wild type *E. coli* K12 W3110) are given in a logarithmic scale as colony forming units (CFU) per µg of pRSF-1b plasmid DNA. For each PIM, the number of spacers integrated in either CRISPR locus 2.1 or 2.3 is given. All spacers have an AAG PAM, unless indicated otherwise. The exact spacer composition of each PIM is given in Table S1.

Upon prolonged cultivation (∼1–2 weeks) at 37°C under non-selective conditions *E. coli* Δ*hns* is cured from the 3.7 kb high copy number plasmid pRSF-1b. Out of 75 individual non-selectively propagated clones tested, 59 (79%) were kanamycin sensitive and 16 (21%) kanamycin resistant. Sequencing of PCR amplicons of CRISPR loci 2.1 and 2.3 showed that between one and five anti-plasmid spacers were integrated in all Kan^S^ clones (Fig. 1, Table S1), while Kan^R^ clones did not contain any new spacers. No plasmid DNA could be isolated from eight out of eight tested Kan^S^ clones (nr. 1, 2, 3, 4, 6, 7, 19, and 27; Fig. 1, Table S1), confirming that the Kan^S^ clones were indeed cured from pRSF-1b. When these clones were retransformed with pRSF-1b a 100- to 1000-fold drop in transformation efficiency was observed for clones with one or two integrated spacers, respectively (Fig. 2). These combined results indicate that the Kan^S^ clones are indeed PIMs. When retransformation efficiencies of PIMs with spacers integrated in either CRISPR 2.1 or 2.3 were compared, no significant differences in efficiencies could be observed, indicating that spacers from both loci are actively transcribed and utilized. Transformation of the PIMs with the target plasmid is not completely inhibited because point mutations in the protospacer at critical positions (seed region or protospacer adjacent motif (PAM) [22]), or deletions, allow pRSF-1b to ‘escape’ the CRISPR interference [9]. This explains why PIMs containing multiple anti-plasmid spacers exhibited lower transformation efficiencies as mutation of multiple protospacers or their PAMs simultaneously occurs at lower frequencies.

Sequencing of the leader-flanking end of CRISPR loci 2.1 and 2.3 of a random selection of 46 Kan^S^ clones revealed a total of 27 unique PIMs carrying a total of 37 different anti-plasmid spacers (Fig. 1, Table S1). While 13 PIMs had integrated a single new spacer, 7, 4, 2 and 1 PIMs integrated two, three, four and five new spacers, respectively. Of all different PIMs, 67% had integrated between one and three spacers in the CRISPR 2.1 locus, while 74% had integrated one or two spacers into the CRISPR 2.3 locus, indicating that both clusters are active.

New spacers were always integrated directly downstream from the leader-flanking repeat. This suggests that there is a specific signal in the leader sequence to integrate new repeat-spacer units at this position in the CRISPR array. No spacer deletion was observed, indicating that the acquisition of new spacers occurs via addition rather than substitution. This is in agreement with findings in *S. thermophilus,* where repeat-spacer units were also mainly added directly downstream of the leader sequence [1], [18], [19]. In agreement with our findings, bioinformatic analyses have shown that spacer turnover and internal spacer integration is a rare event in *E. coli* [5], [23].

### AAG is the Dominant Protospacer Adjacent Motif

The protospacer adjacent motif (PAM) is a short conserved nucleotide sequence located in a protospacer flanking region [22]. The analysis of spacer-protospacer pairs from over 150 species has revealed the existence of several PAM consensus sequences which co-occur with specific repeat types [22]. The PAM consensus sequence 5′-AWG-protospacer-3′ was identified for *E. coli* [22]. When present, PAMs are essential for CRISPR-interference as a point mutation in the PAM allows bacteriophages to escape the immune system [9], [24]. For *E. coli* it was shown that mutations in the PAM result in dramatically lower target DNA binding affinity of the crRNA guided complex Cascade [9], explaining how the bacteriophage genome can avoid being detected.

Of all integrated spacers, 29 (78%) corresponded to protospacers with an AAG PAM, one (3%) with an ATG PAM, and seven (19%) with non-consensus PAM sequences (AAA, AGG (2x), GAG, TAG, CGA, AAT; Table S1). Although the functionality of only the ATG PAM has been verified in *E. coli* [9], the majority of integrated spacers in our experiments correspond to protospacers flanking an AAG PAM. It could be argued that spacers are selected randomly followed by natural selection. Clones that have integrated spacers with a consensus PAM (AWG) are cured from the high copy number plasmid pRSF-1b and generally gain an energetic growth advantage [25], which allows them to outgrow clones that have incorporated spacers with non-functional PAMs. However, this would have resulted in a more equal distribution of AAG and ATG PAMs, making the random spacer selection process unlikely. Furthermore, since an AAG triplets are found less frequently on pRSF-1b than ATG triplets (94 times AAG versus 129 times ATG), limited availability ATG is not the reason for AAG PAM selection. Moreover, five spacers were integrated multiple times in unrelated PIMs and in different CRISPR loci (S4 in PIM 20 (2×) and 25; S8 in PIM 5, 9, 17 and 25; S12 in PIM 10, 11 and 18; S33 in PIM 14 and 23; S34 in PIM 17, 22 and 25) which also argues against random spacer selection. These findings indicate that there is a selection for AAG PAM sequences during spacer acquisition.

It is worth noting that three PIMs (4, 19, and 27) integrated a single anti-plasmid spacer corresponding to the non-PAM consensus sequences AGG, GAG and AAT. Sequencing of pRSF-1b in the corresponding regions excluded the possibility that the plasmid contained mutations at these positions, confirming that these PAMs were indeed non-consensus PAM sequences. The fact that these PIMs were cured from the plasmid, and were less susceptible to retransformation of the target plasmid (Fig. 2) indicates that these non-consensus PAMs are additionally allowed during CRISPR interference. PIM 1, which integrated a spacer with a non-consensus AAA PAM and one other spacer, shows resistance typical for PIMs with two functional spacers. This indicates that also this PAM is likely to be allowed during CRISPR interference.

Interestingly, the AAT-PAM spacer S16 in PIM 27 which targets the kanamycin resistance gene provides higher resistance to retransformation with the target plasmid than single spacers in PIM 4 and 19 targeting the same gene (Fig. 2). This can be explained by the fact that this spacer targets a relatively well conserved region of the kanamycin resistance gene encoding Glu68 [26], [27]. Mutation of the S16 protospacer may therefore result in more frequent loss of Kanamycin nucleotidyl transferase activity.

**Figure 3:**
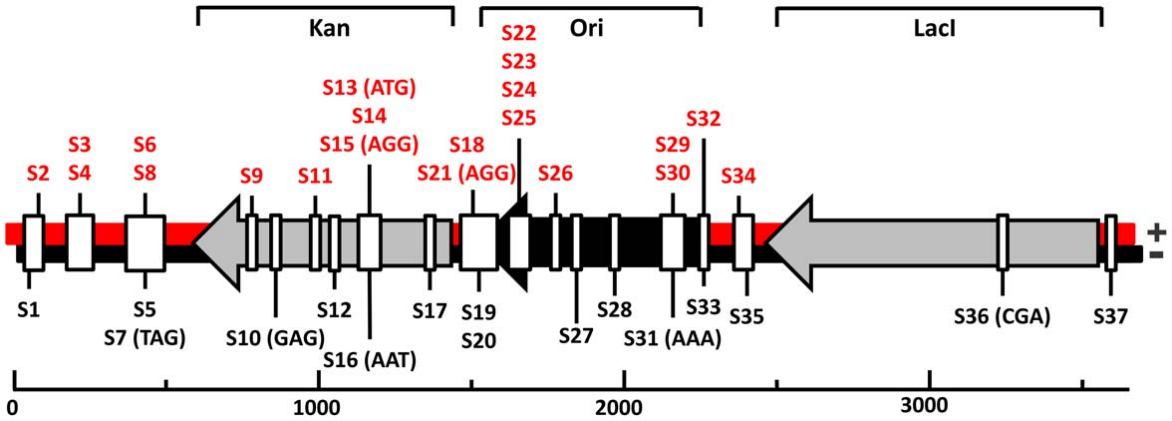
Linear display of pRSF-1b and locations of protospacers. The (+) and (−) strands and corresponding protospacers are coloured red and black, respectively. Kanamycin marker (Kan), Origin of replication (Ori) and *lacI* (LacI) are shown as arrows. Protospacers have an AAG PAM unless indicated otherwise.

### Counterselection for Self-targeting Spacers

The locations of the protospacers were mapped on both strands of the plasmid (43% and 57% on the (+) and (−) strand, respectively) and covered regions of the backbone and multiple cloning site (32%), origin of replication (40%) and the kanamycin resistance gene (24%) (Fig.3). This indicates that protospacer acquisition occurs independently of transcription or direction of replication of the plasmid. Interestingly, only a single spacer (2%) was integrated against the plasmid-encoded *lacI* gene (S36; Fig. 3). This observation can be explained by the presence of a nearly identical copy (one nucleotide difference) of the *lacI* gene in the *E. coli* K12 genome. Spacers targeting the plasmid encoded *lacI* gene would therefore also target the *E. coli* genome, leading to lethal DNA damage, and resulting in a counterselection for these variants. This result fits very well with the observation that spacers against a prophage are lethal to *E. coli* [8]. The identified anti-*lacI* spacer in PIM 23 has a non-consensus PAM CGA that possibly prevents self-targeting. The plasmid interfering phenotype of this PIM is likely to be caused by the two additional spacers corresponding to protospacers with AAG PAMs (Fig. 1, Table S1).

### Nucleotide Composition of Spacers

**Figure 4:**
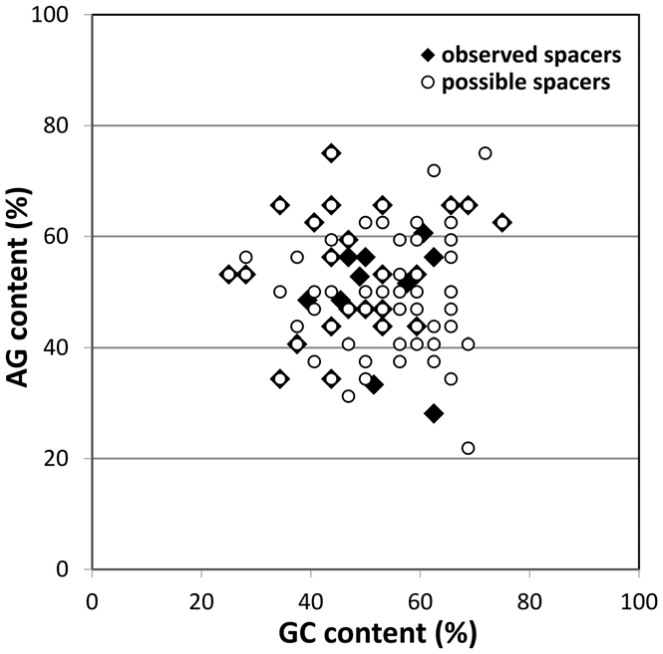
Graphical representation of AG and GC contents of each observed and possible spacer. Observed spacers (⧫) are spacers integrated in CRISPR loci 2.1 and 2.3 (Table S1). These spacers are 32 or 33-mers with various PAMs. Possible spacers (Ο) are all 32-mers found on pRSF-1b directly downstream of an AAG PAM.

The nucleotide content of the 37 unique anti-plasmid spacers was compared with the composition of all possible AAG-flanking protospacers on pRSF-1b (Fig. 4). The analysis showed that the integrated spacers displayed no selection bias for GC-content. This suggests that GC content of the protospacers, and therefore the local stability of the DNA duplex, plays no major role during spacer selection. In addition to GC content, we also analysed purine (AG) content of the new spacers (Fig. 4), as purine-rich RNA is known to basepair energetically more favourable with DNA than the corresponding DNA:DNA duplex [28], [29]. This may be of importance during the hybridization of the crRNA to double stranded target DNA molecules. Again, no apparent bias could be observed compared to the semi-randomly generated spacer set, suggesting that the energetic gain of pairing purine rich crRNA with DNA by Cascade is not taken into account by the CRISPR adaptation machinery during spacer integration. Also no bias was found for GC or AG-content in the seed sequence, which plays an important role in during target DNA binding of Cascade [9].

### The Last Nucleotide of the Repeat is PAM Derived

It has previously been described that repeats of CRISPR 2.1 and 2.3 (consensus: 5′-GWGTTCCCCGCGCCAGCGGGGATAAACCG-3′) contain polymorphisms [5]. Some polymorphisms in the repeats have been associated with preventing self-targeting, as self-targeting spacers are often accompanied by degraded repeats [30]. Especially the last 8 nucleotides of the repeat, which determine the first 8 nucleotides of mature crRNAs, appear to be important for the functioning of CRISPR/Cas systems [4]. The Type III-a system of *Staphylococcus epidermidis* uses differential complementarity of these first 8 nucleotides of the crRNA with one protospacer flank to discriminate between self DNA (the CRISPR) and non-self DNA (the target), preventing autoimmunity [31]. Other CRISPR/Cas systems may use PAMs to determine if a sequence will be targeted [9], [18], [19], [22].

**Figure 5:**
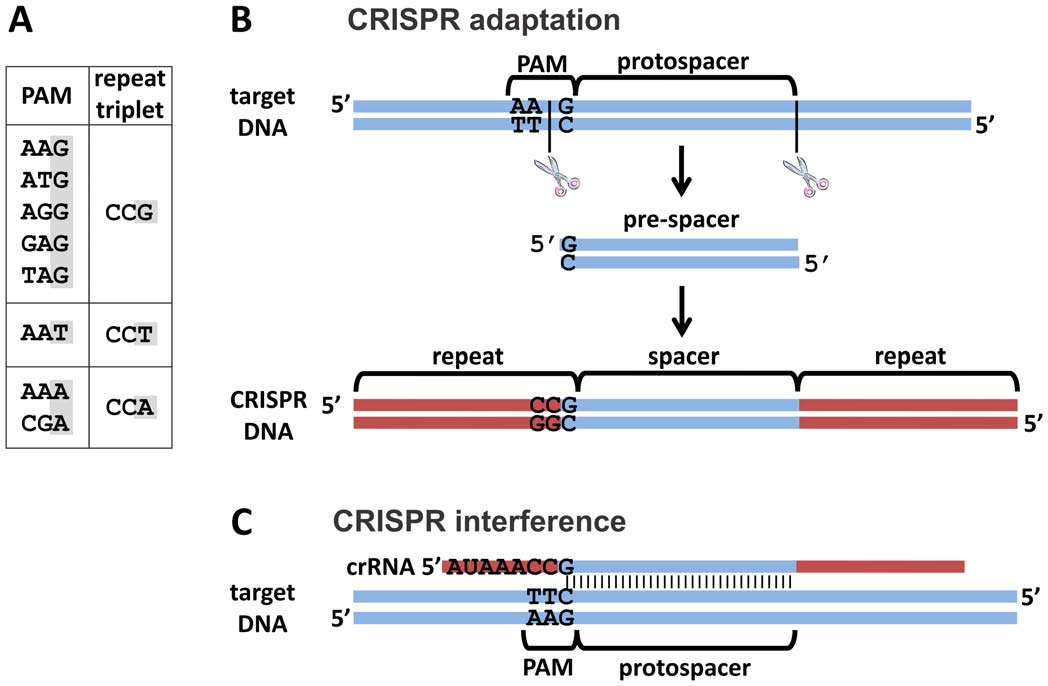
PAM and repeat-end correlation. (A): PAMs of observed spacers and the co-occurring trinucleotide repeat-ends associated with these spacers. Notice that the spacer-proximal nucleotide of the repeat end is identical to the protospacer-proximal nucleotide of the PAM. (B): Schematic of the proposed mechanism for spacer acquisition during CRISPR adaptation. A protospacer with specific PAM is selected after which it is processed into the pre-spacer (at least 33–34 bp), which contains the last nucleotide of the PAM (the pre-spacer could be single-stranded or double-stranded). The pre-spacer is than integrated at the leader proximal end of the CRISPR locus. The nucleotide derived from the PAM forms the last nucleotide of the repeat. (C): R-loop formation by mature crRNA (61 nucleotides) during CRISPR interference. Notice that the last nucleotide of the repeat (the nucleotide derived from the PAM) is complementary to the target DNA sequence. It remains unknown whether base-pairing between these nucleotides is important for interference.

Our dataset shows that the last three nucleotides of the repeat (CCG) occasionally carry mutations. Repeat 2 of CRISPR locus 2.3 in the parental strain contains a polymorphism at the last nucleotide, changing the trinucleotide sequence from CCG to CCT. Almost all PIMs with new spacers in CRISPR 2.3, however, did not carry this polymorphism in their new repeats, indicating that the second repeat in a CRISPR is not duplicated during the spacer integration process.

S16 is preceded by a CCT trinucleotide repeat sequence, and strikingly this spacer corresponds to a protospacer with non-consensus AAT PAM. This combination is apparently functional, as this PIM is cured from the plasmid and is less susceptible for retransformation with pRSF-1b (Fig. 2), while carrying only one anti-plasmid spacer. This indicates that S16 facilitates interference although it has a non-consensus PAM and a mutated repeat.

S31 in PIM 1 is preceded by a CCA trinucleotide repeat sequence and it has the non-consensus AAA PAM, while spacer S36 in PIM 23 is preceded by repeat sequence CCA and targets a plasmid sequence flanking a non-consensus CGA PAM. Because PIM 1 and 23 each contain additional typical anti-plasmid spacers, it cannot be concluded whether S31 and S23 are functional. However PIM 1 (carrying S31 and typical spacer S17) shows a decrease in transformation efficiency similar to PIMs with two typical anti-plasmid spacers (Fig. 2), suggesting that S17 is indeed functional.

Interestingly, the last nucleotide of the repeat preceding the new spacer always matched the third nucleotide of the PAM, both in normal situations (repeat CCG, and AAG, match underlined) and in deviations from normal (CCT^R^ – AAT^P^; CCA^R^ – CGA^P^; CCA^R^ – AAA^P^; Fig. 5A). The single nucleotide polymorphism (SNP) at the last position of the repeat and corresponding deviations from the PAM consensus sequence suggests that the last nucleotide of the repeat is derived from the PAM in the target DNA (Fig. 5B). Evidence supporting this hypothesis is provided in PIMs 1 and 23 which contain the deviated repeat-spacer unit at the second position in the locus and have a consensus repeat-spacer unit at the first position. Apparently, the repeat SNP is not propagated in the new repeat-spacer unit at the first position in the locus (Table S1, PIM1 and PIM23), but reverted to the repeat-consensus by the selection of a normal AAG PAM-containing protospacer. We hypothesize that the protospacer-flanking nucleotide of the PAM is still attached to the selected, to-be-integrated spacer (pre-spacer [32]), and forms the last nucleotide of the proximal repeat after integration is complete (Fig. 5B). As a consequence, this nucleotide in the crRNA is always complementary to the protospacer-flanking nucleotide of the PAM (Fig. 5C), even when a non-consensus PAM is selected during spacer acquisition.

### Spacer Integration Patterns Suggest a Positive Feedback Loop of Active Spacers

**Figure 6:**
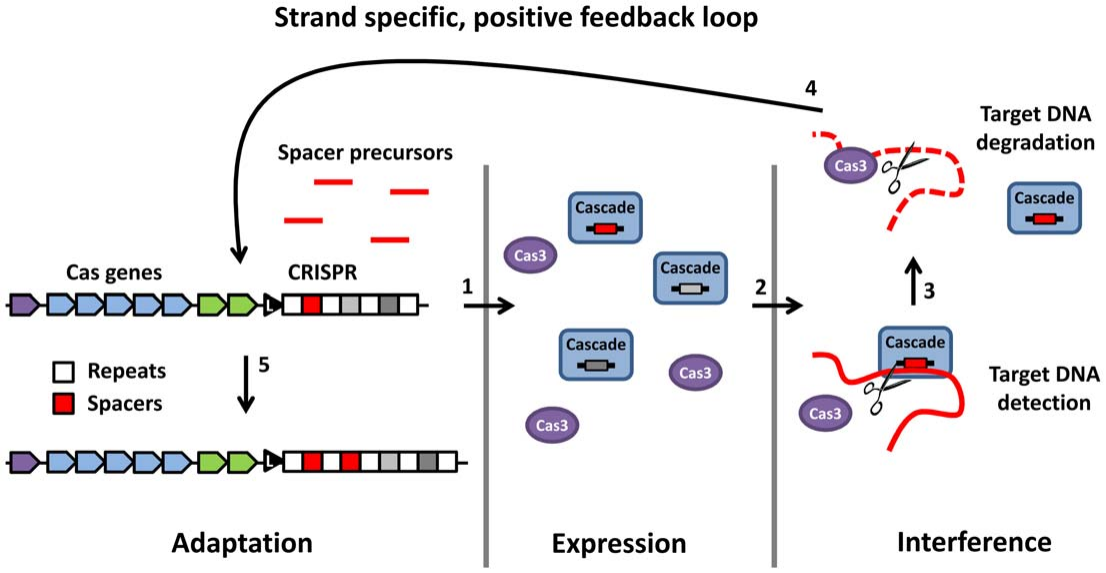
Model of the strand specific positive feedback loop. Cells with a spacer against a known and actively present invader DNA produce targeting Cascade complexes in the expression stage. In the interference stage, Cascade binds the target dsDNA after which the target is cleaved and degraded by Cas3 [15]. DNA degradation products generated by Cascade and Cas3 (which could be ssDNA or dsDNA) act as precursors for new spacers in the adaptation phase in a strand-specific manner. By integration of these strand-specific precursors, the spacer repertoire against an actively present invader is expanded, completing the positive feedback loop.

In 14 different PIMs, two or more spacers were integrated (Fig. 1, Table S1). No preference for a specific target location of subsequently integrated spacers could be detected, such as a location near the target site of the primary integrated spacer. However, all spacers of an individual PIM always targeted the same strand of the plasmid, implying that the primary integrated spacer determines which strand subsequently integrated spacers will target. This suggests a positive feedback loop that may result from interplay between the CRISPR interference machinery (Cascade and Cas3) and the spacer integration machinery. We hypothesize that CRISPR-mediated plasmid degradation by Cas3 [15], guided by crRNA from an active spacer – the first new spacer in this case - generates specific DNA degradation products that are used as precursors for subsequent new spacers (Fig. 6). These findings are in contrast with new spacer integration patterns in *S. thermophilus*, where secondary spacers show no strand selection bias [1], [18], [33] suggesting that CRISPR acquisition and CRISPR interference by Cas9 [34] are independent processes in *S. thermophilus*.

### Conclusions

*E. coli* K12 is cured from a high copy number plasmid by integrating anti-plasmid spacers in two of its CRISPR loci. New spacers are selected in a non-random process that takes into account the presence of a PAM on the target DNA. We hypothesize that the mechanism of CRISPR adaptation in Type I-E systems involves selection of protospacers including one nucleotide from the PAM, which determines the last nucleotide of the preceding repeat. Spacer analysis further suggests a positive feedback loop between active spacers in a cluster and newly acquired spacers, through interplay of the CRISPR interference and adaptation machinery. Possibly the target DNA degradation products generated by Cascade and Cas3 serve as precursors for the integration of new spacers against the same target (Fig. 6). Increasing the number of spacers targeting an invading DNA element may represent an efficient strategy to expand the repertoire of spacers targeting a specific invader to amplify the CRISPR interference effect. Having multiple active spacers against the same target reduces the chance that invaders evade immunity by point mutation in the seed region of the protospacer or PAM, since point mutations at multiple target sites simultaneously occur at lower frequencies.

## Materials and Methods

### Plasmid Curing

*Escherichia coli* K12 W3110 derivate Δ*hns* (JW1225) from the KEIO collection [35] was supplied by the American Type Culture Collection (ATCC). Its kanamycin resistance marker was removed according to protocol described by Datsenko *et al* [36]. This strain was transformed with high copy number plasmid pRSF-1b (Novagen) (RSF1030 origin of replication, >100 copies/cell, 3.7 kb [37]) as described below. Colonies were picked from an LB-agar plate containing 100 µg/mL kanamycin and used to inoculate 2YTL medium [7] containing no antibiotics. The culture was transferred daily to fresh 2YTL medium in a shaking incubator for prolonged periods of time (∼1–2 weeks). The culture was regularly checked for plasmid loss by plating on non-selective LB-plates, followed by replica streaking on selective and non-selective plates.

### Transformations

Cells for the plasmid curing experiments and retransformation experiments were made chemically competent using the RuCl method and transformed by applying a heat-shock as described in the QIAexpressionist handbook (QIAGEN). After transformation, cells were plated on an LB-agar plate containing 100 µg/mL kanamycin.

### Colony PCR

Clones were screened for spacer integration by colony PCR using DreamTaq Green DNA polymerase (Fermentas). New spacers in the CRISPR 2.1 locus were PCR amplified using forward primer BG3474 (′5-AAATGTTACATTAAGGTTGGTG-′3) annealing 72 bases upstream of the first repeat and reverse primer BG3475 (′5-GAAATTCCAGACCCGATCC-′3) annealing in spacer 4 of this locus. New spacers in the CRISPR 2.3 locus were PCR amplified using forward primer BG3414 (′5-GGTAGATTTTAGTTTGTATAGAG-′3) annealing 164 bases upstream of the first repeat and BG3415 (′5-CAACAGCAGCACCCATGAC-′3) annealing in spacer 3 of this locus. PCR product sizes were estimated using agarose gels and SYBR-safe DNA gel stain (Invitrogen). The CRISPR 2.1 and 2.3 loci of 46 clones Kan^S^ clones were sequenced by GATC-Biotech (Konstanz, Germany) with BG3474 and BG3414, respectively.

### Spacer Composition Analysis

Nucleotide analyses were carried out using in-house perl scripts. In brief, all 32-mers from plasmid pRSF-1b preceded by the PAM AAG were tested for their nucleotide composition, and compared to the nucleotide composition of all experimentally retrieved spacers.

### Plasmid Loss Studies

PIM 1, 2, 3, 4, 6, 7, 19 and 27 were cultured in 5 ml LB medium without antibiotics, and were incubated o/n in a rotary shaker at 37°C. The o/n cultures were miniprepped (GeneJET, Fermentas) and the absence of plasmid DNA in the eluate was verified by nanodrop and agarose gel electrophoresis. The same PIMs and the wild-type control strain were retransformed with pRSF-1b and plated on LB-agar plates containing 50 µg/ml Kan. Transformations efficiency was determined as the number of colony forming units per µg plasmid DNA.

## Supporting Information

Table S1 Integrated spacer sequences. PIM: Plasmid interfering mutant, Spacer #: spacer number, corresponds to spacer locations in Fig. 1 and Fig. 3. PAM: protospacer adjacent motif, non-consensus PAMs are underlined. Target: Location of protospacer on pRSF-1b (Kan: Kanamycin resistance gene. Ori: RSF1030 origin of replication. Bb: pRSF-1b backbone, lacI: lac operon repressor gene). Target position: Nucleotide position of the spacer match (protospacer) on pRSF-1b. Spacers matching protospacers on the (+) and (-) strand are coloured red and black, respectively. Note that the actual targeted strand during CRISPR interference by Cascade is the complementary strand of what is indicated here.
(DOCX)

## References

[1] Barrangou R, Fremaux C, Deveau H, Richards M, Boyaval P (2007). CRISPR provides acquired resistance against viruses in prokaryotes.. Science.

[2] Karginov FV, Hannon GJ (2010). The CRISPR system: small RNA-guided defense in bacteria and archaea.. Molecular cell.

[3] Horvath P, Barrangou R (2010). CRISPR/Cas, the immune system of bacteria and archaea.. Science.

[4] Jore MM, Brouns SJ, van der Oost J (2011). RNA in Defense: CRISPRs Protect Prokaryotes against Mobile Genetic Elements.. http://dx.doi.org/10.1101/cshperspect.a003657.

[5] Diez-Villasenor C, Almendros C, Garcia-Martinez J, Mojica FJ (2010). Diversity of CRISPR loci in Escherichia coli.. Microbiology.

[6] Makarova KS, Haft DH, Barrangou R, Brouns SJ, Charpentier E (2011). Evolution and classification of the CRISPR-Cas systems.. Nature reviews Microbiology.

[7] Brouns SJ, Jore MM, Lundgren M, Westra ER, Slijkhuis RJ (2008). Small CRISPR RNAs guide antiviral defense in prokaryotes.. Science.

[8] Edgar R, Qimron U (2010). The Escherichia coli CRISPR system protects from lambda lysogenization, lysogens, and prophage induction.. Journal of bacteriology.

[9] Semenova E, Jore MM, Datsenko KA, Semenova A, Westra ER (2011). Interference by clustered regularly interspaced short palindromic repeat (CRISPR) RNA is governed by a seed sequence.. Proceedings of the National Academy of Sciences of the United States of America.

[10] Touchon M, Rocha EP (2010). The small, slow and specialized CRISPR and anti-CRISPR of Escherichia and Salmonella.. PLoS One.

[11] Pul U, Wurm R, Arslan Z, Geissen R, Hofmann N (2010). Identification and characterization of E. coli CRISPR-cas promoters and their silencing by H-NS.. Molecular microbiology.

[12] Jore MM, Lundgren M, van Duijn E, Bultema JB, Westra ER (2011). Structural basis for CRISPR RNA-guided DNA recognition by Cascade.. Nature structural & molecular biology.

[13] Sinkunas T, Gasiunas G, Fremaux C, Barrangou R, Horvath P (2011). Cas3 is a single-stranded DNA nuclease and ATP-dependent helicase in the CRISPR/Cas immune system.. The EMBO journal.

[14] Howard JA, Delmas S, Ivancic-Bace I, Bolt EL (2011). Helicase dissociation and annealing of RNA-DNA hybrids by Escherichia coli Cas3 protein.. The Biochemical journal.

[15] Westra ER, Van Erp PBG, Künne T, Wong SP, Staals R (2012). CRISPR immunity relies on the consecutive binding and degradation of negatively supercoiled invader DNA by Cascade and Cas3..

[16] Pougach K, Semenova E, Bogdanova E, Datsenko KA, Djordjevic M (2010). Transcription, processing and function of CRISPR cassettes in Escherichia coli.. Molecular microbiology.

[17] Westra ER, Pul U, Heidrich N, Jore MM, Lundgren M (2010). H-NS-mediated repression of CRISPR-based immunity in Escherichia coli K12 can be relieved by the transcription activator LeuO.. Molecular microbiology.

[18] Horvath P, Romero DA, Coute-Monvoisin AC, Richards M, Deveau H (2008). Diversity, activity, and evolution of CRISPR loci in Streptococcus thermophilus.. Journal of bacteriology.

[19] Garneau JE, Dupuis ME, Villion M, Romero DA, Barrangou R (2010). The CRISPR/Cas bacterial immune system cleaves bacteriophage and plasmid DNA.. Nature.

[20] Nam KH, Kurinov I, Ke A (2011). Crystal structure of clustered regularly interspaced short palindromic repeats (CRISPR)-associated Csn2 protein revealed Ca2+-dependent double-stranded DNA binding activity.. The Journal of biological chemistry.

[21] van der Oost J, Jore MM, Westra ER, Lundgren M, Brouns SJ (2009). CRISPR-based adaptive and heritable immunity in prokaryotes.. Trends in biochemical sciences.

[22] Mojica FJ, Diez-Villasenor C, Garcia-Martinez J, Almendros C (2009). Short motif sequences determine the targets of the prokaryotic CRISPR defence system.. Microbiology.

[23] Touchon M, Charpentier S, Clermont O, Rocha EP, Denamur E (2011). CRISPR distribution within the Escherichia coli species is not suggestive of immunity-associated diversifying selection.. Journal of bacteriology.

[24] Deveau H, Barrangou R, Garneau JE, Labonte J, Fremaux C (2008). Phage response to CRISPR-encoded resistance in Streptococcus thermophilus.. Journal of bacteriology.

[25] Dykhuizen DE, Hartl DL (1983). Selection in chemostats.. Microbiol Rev.

[26] Martin P, Jullien E, Courvalin P (1988). Nucleotide sequence of Acinetobacter baumannii aphA-6 gene: evolutionary and functional implications of sequence homologies with nucleotide-binding proteins, kinases and other aminoglycoside-modifying enzymes.. Mol Microbiol.

[27] Nurizzo D, Shewry SC, Perlin MH, Brown SA, Dholakia JN (2003). The crystal structure of aminoglycoside-3′-phosphotransferase-IIa, an enzyme responsible for antibiotic resistance.. J Mol Biol.

[28] Hall KB, McLaughlin LW (1991). Thermodynamic and structural properties of pentamer DNA.DNA, RNA.RNA, and DNA.RNA duplexes of identical sequence.. Biochemistry.

[29] Roberts RW, Crothers DM (1992). Stability and properties of double and triple helices: dramatic effects of RNA or DNA backbone composition.. Science.

[30] Stern A, Keren L, Wurtzel O, Amitai G, Sorek R (2010). Self-targeting by CRISPR: gene regulation or autoimmunity?. Trends in genetics.

[31] Marraffini LA, Sontheimer EJ (2010). Self versus non-self discrimination during CRISPR RNA-directed immunity.. Nature.

[32] Al-Attar S, Westra ER, van der Oost J, Brouns SJ (2011). Clustered regularly interspaced short palindromic repeats (CRISPRs): the hallmark of an ingenious antiviral defense mechanism in prokaryotes.. Biological chemistry.

[33] van der Ploeg JR (2009). Analysis of CRISPR in Streptococcus mutans suggests frequent occurrence of acquired immunity against infection by M102-like bacteriophages.. Microbiology.

[34] Sapranauskas R, Gasiunas G, Fremaux C, Barrangou R, Horvath P (2011). The Streptococcus thermophilus CRISPR/Cas system provides immunity in Escherichia coli.. http://dx.doi.org/10.1093/nar/gkr606.

[35] Baba T, Huan HC, Datsenko K, Wanner BL, Mori H (2008). The applications of systematic in-frame, single-gene knockout mutant collection of Escherichia coli K-12.. Methods in molecular biology.

[36] Datsenko KA, Wanner BL (2000). One-step inactivation of chromosomal genes in Escherichia coli K-12 using PCR products.. Proceedings of the National Academy of Sciences of the United States of America.

[37] Conrad SE, Wold M, Campbell JL (1979). Origin and direction of DNA replication of plasmid RSF1030.. Proceedings of the National Academy of Sciences of the United States of America.

