# Supplementary material for: CRISPR Interference Directs Strand Specific Spacer Acquisition
Source: PLoS One. 2012 Apr 27;7(4):e35888. doi: 10.1371/journal.pone.0035888 (PMC3338789; doi:10.1371/journal.pone.0035888)
Supplement: Table S1 — Integrated spacer sequences. PIM: Plasmid interfering mutant, Spacer #: spacer number, corresponds to spacer locations in Fig. 1 and Fig. 3. PAM: protospacer adjacent motif, non-consensus PAMs are underlined. Target: Location of protospacer on pRSF-1b (Kan: Kanamycin resistance gene. Ori: RSF1030 origin of replication. Bb: pRSF-1b backbone, lacI: lac operon repressor gene). Target position: Nucleotide position of the spacer match (protospacer) on pRSF-1b. Spacers matching protospacers on the (+) and (-) strand are coloured red and black, respectively. Note that the actual targeted strand during CRISPR interference by Cascade is the complementary strand of what is indicated here. (DOCX) [file pone.0035888.s001.docx]

**Table S1: Integrated spacer sequences**

| **PIM** | **CRISPR locus** | **Spacer**  **position** | **Spacer #** | **Repeat sequence (5’ to 3’)**  **(located directly upstream of spacer sequence)** | **Spacer sequence (5’ to 3’)** | **Spacer**  **length (nt)** | **PAM** | **Target** | **Target**  **Position**  **(nt)** | **Target**  **plasmid**  **strand** |
| --- | --- | --- | --- | --- | --- | --- | --- | --- | --- | --- |
| 1  1 | 2.1 | -2 | S17 | GAGTTCCCCGCGCCAGCGGGGATAAACCG | AGTATGAGCCATATTCAACGGGAAACGTCTTG | 32 | AAG | Kan | 1366 | - |
|  | 2.1 | -1 | S31 | GAGTTCCCCGCGCCAGCGGGGATAAACCA | GAAAAACCACCGCTACCAACGGTGGTTTTCTCA | 33 | AAA | Ori | 2161 | - |
| 2 | 2.1 | -1 | S22 | GAGTTCCCCGCGCCAGCGGGGATAAACCG | CCGGAAAGAACATGTGAGCAAAAAGCAAAGCA | 32 | AAG | Ori | 1613 | + |
| 2 | 2.3 | -1 | S11 | GTGTTCCCCGCGCCAGCGGGGATAAACCG | GACAATTACAAACAGGAATCGAATGCAACCGG | 32 | AAG | Kan | 977 | + |
| 3 | 2.3 | -2 | S32 | GTGTTCCCCGCGCCAGCGGGGATAAACCG | TCAACGAACAGCTATTCCGTTACTCTAGATTT | 32 | AAG | Ori | 2231 | + |
| 3 | 2.3 | -1 | S23 | GTGTTCCCCGCGCCAGCGGGGATAAACCG | AACATGTGAGCAAAAAGCAAAGCACCGGAAGA | 32 | AAG | Ori | 1621 | + |
| 4 | 2.1 | -1 | S15 | GAGTTCCCCGCGCCAGCGGGGATAAACCG | CATAAATTCCGTCAGCCAGTTTAGTCTGACCA | 32 | AGG | Kan | 1158 | + |
| 5 | 2.1 | -1 | S8 | GAGTTCCCCGCGCCAGCGGGGATAAACCG | CTGACGACCGGGTCTCCGCAAGTGGCACTTTT | 32 | AAG | Bb | 469 | + |
| 5 | 2.3 | -1 | S3 | GTGTTCCCCGCGCCAGCGGGGATAAACCG | CTTGCGGCCGCACTCGAGTCTGGTAAAGAAACC | 33 | AAG | Bb | 182 | + |
| 6 | 2.1 | -1 | S35 | GAGTTCCCCGCGCCAGCGGGGATAAACCG | TTAGcTcactCATTAGGCACCGGGATCTCGAC | 32 | AAG | Bb | 2394 | - |
| 7 | 2.3 | -1 | S2 | GTGTTCCCCGCGCCAGCGGGGATAAACCG | GAGATATACCATGGCACATCACCACCACCATC | 32 | AAG | Bb | 61 | + |
| 8 | 2.3 | -1 | S5 | GTGTTCCCCGCGCCAGCGGGGATAAACCG | CAGTGTGACCGTGTGCTTCTCAAATGCCTGAG | 32 | AAG | Bb | 357 | - |
| 9 | 2.1 | -2 | S14 | GAGTTCCCCGCGCCAGCGGGGATAAACCG | AGGCATAAATTCCGTCAGCCAGTTTAGTCTGAC | 33 | AAG | Kan | 1155 | + |
| 9 | 2.1 | -1 | S21 | GAGTTCCCCGCGCCAGCGGGGATAAACCG | CATGCAGCGCTCTTCCGCTTCCTCGCTCACTG | 32 | AGG | Ori | 1497 | + |
| 9 | 2.3 | -1 | S8 | GTGTTCCCCGCGCCAGCGGGGATAAACCG | CTGACGACCGGGTCTCCGCAAGTGGCACTTTT | 32 | AAG | Bb | 469 | + |
| 10 | 2.3 | -1 | S12 | GTGTTCCCCGCGCCAGCGGGGATAAACCG | AATATCCTGATTCAGGTGAAAATATTGTTGAT | 32 | AAG | Kan | 1029 | - |
| 11 | 2.1 | -2 | S20 | GAGTTCCCCGCGCCAGCGGGGATAAACCG | CGGAAGAGCGCTGCATGCCTATTTGTTTATTT | 32 | AAG | Ori | 1476 | - |
| 11 | 2.1 | -1 | S12 | GAGTTCCCCGCGCCAGCGGGGATAAACCG | AATATCCTGATTCAGGTGAAAATATTGTTGAT | 32 | AAG | Kan | 1029 | - |
| 11 | 2.3 | -1 | S5 | GTGTTCCCCGCGCCAGCGGGGATAAACCG | CAGTGTGACCGTGTGCTTCTCAAATGCCTGAG | 32 | AAG | Bb | 357 | - |
| 12 | 2.1 | -1 | S37 | GAGTTCCCCGCGCCAGCGGGGATAAACCG | GGAGAGCGTCGAGATCCCGGACACCATCGAATG | 33 | AAG | Bb | 3594 | - |
| 13 | 2.1 | -1 | S1 | GAGTTCCCCGCGCCAGCGGGGATAAACCG | TTAAACAAAATTATTTCTACAGGGGAATTGTT | 32 | AAG | Bb | 19 | - |
| 14 | 2.1 | -1 | S33 | GAGTTCCCCGCGCCAGCGGGGATAAACCG | AGATAAATTGCACTGAAATCTAGAGTAACGGA | 32 | AAG | Bb | 2246 | - |
| 14 | 2.3 | -1 | S5 | GTGTTCCCCGCGCCAGCGGGGATAAACCG | CAGTGTGACCGTGTGCTTCTCAAATGCCTGAG | 32 | AAG | Bb | 357 | - |
| 15 | 2.1 | -1 | S24 | GAGTTCCCCGCGCCAGCGGGGATAAACCG | CAAAGCACCGGAAGAAGCCAACGCCGCAGGCG | 32 | AAG | Ori | 1638 | + |
| 15 | 2.3 | -1 | S6 | GTGTTCCCCGCGCCAGCGGGGATAAACCG | CACACGGTCACACTGCTTCCGGTAGTCAATAA | 32 | AAG | Bb | 374 | + |
| 16 | 2.3 | -1 | S27 | GTGTTCCCCGCGCCAGCGGGGATAAACCG | CGGAAGAGCGCTGCATGCCTATTTGTTTATTT | 32 | AAG | Ori | 1476 | - |
| 17 | 2.1 | -2 | S8 | GAGTTCCCCGCGCCAGCGGGGATAAACCG | CTGACGACCGGGTCTCCGCAAGTGGCACTTTT | 32 | AAG | Bb | 469 | + |
| 17 | 2.1 | -1 | S34 | GAGTTCCCCGCGCCAGCGGGGATAAACCG | TTGTAATTCTCATGTTAGTCATGCCCCGCGCCC | 33 | AAG | Bb | 2319 | + |
| 17 | 2.3 | -2 | S6 | GTGTTCCCCGCGCCAGCGGGGATAAACCG | CACACGGTCACACTGCTTCCGGTAGTCAATAA | 32 | AAG | Bb | 374 | + |
| 17 | 2.3 | -1 | S26 | GTGTTCCCCGCGCCAGCGGGGATAAACCG | CCAGAGGTGGCGAAACCCGACAGGACTATAAA | 32 | AAG | Ori | 1723 | + |

| **PIM** | **CRISPR locus** | **Spacer**  **position** | **Spacer**  **#** | **Repeat sequence (5’ to 3’)**  **(located directly upstream of spacer sequence)** | **Spacer sequence (5’ to 3’)** | **Spacer**  **length (nt)** | **PAM** | **Target** | **Target**  **Position**  **(nt)** | **Target**  **plasmid**  **strand** |
| --- | --- | --- | --- | --- | --- | --- | --- | --- | --- | --- |
| 18 | 2.1 | -2 | S19 | GAGTTCCCCGCGCCAGCGGGGATAAACCG | AGCGctgcatGCCTATTTGTTTATTTTTCTAA | 32 | AAG | Bb | 1470 | - |
| 18 | 2.1 | -1 | S7 | GAGTTCCCCGCGCCAGCGGGGATAAACCG | CCGCTTATGTCTATTGCTGGTTTACCGGTTTA | 32 | TAG | Bb | 403 | - |
| 18 | 2.3 | -1 | S12 | GTGTTCCCCGCGCCAGCGGGGATAAACCG | AATATCCTGATTCAGGTGAAAATATTGTTGAT | 32 | AAG | Kan | 1029 | - |
| 19 | 2.1 | -1 | S10 | GAGTTCCCCGCGCCAGCGGGGATAAACCG | CGTAATGGCTGGCCTGTTGAACAAGTCTGGAA | 32 | GAG | Kan | 856 | - |
| 20 | 2.1 | -2 | S4 | GAGTTCCCCGCGCCAGCGGGGATAAACCG | AAACCGCTGCTGCGAAATTTGAACGCCAGCAC | 32 | AAG | Bb | 210 | + |
| 20 | 2.1 | -1 | S4 | GAGTTCCCCGCGCCAGCGGGGATAAACCG | AAACCGCTGCTGCGAAATTTGAACGCCAGCAC | 32 | AAG | Bb | 210 | + |
| 20 | 2.3 | -2 | S9 | GTGTTCCCCGCGCCAGCGGGGATAAACCG | GTTATCAAGTGAGAAATCACCATGAGTGACGA | 32 | AAG | Kan | 786 | + |
| 20 | 2.3 | -1 | S18 | GTGTTCCCCGCGCCAGCGGGGATAAACCG | CATTTATCAGGGTTATTGTCTCATGAGCGGATA | 33 | AAG | Bb | 1422 | + |
| 21 | 2.3 | -1 | S25 | GTGTTCCCCGCGCCAGCGGGGATAAACCG | CACCGGAAGAAGCCAACGCCGCAGGCGTTTTT | 32 | AAG | Ori | 1643 | + |
| 22 | 2.3 | -1 | S34 | GTGTTCCCCGCGCCAGCGGGGATAAACCG | TTGTAATTCTCATGTTAGTCATGCCCCGCGCCC | 33 | AAG | Bb | 2319 | + |
| 23 | 2.1 | -1 | S35 | GAGTTCCCCGCGCCAGCGGGGATAAACCG | TTAGcTcactCATTAGGCACCGGGATCTCGAC | 32 | AAG | Bb | 2394 | - |
| 23 | 2.3 | -2 | S33 | GTGTTCCCCGCGCCAGCGGGGATAAACCG | AGATAAATTGCACTGAAATCTAGAGTAACGGA | 32 | AAG | Bb | 2246 | - |
| 23 | 2.3 | -1 | S36 | GTGTTCCCCGCGCCAGCGGGGATAAACCA | TCAACTGGGTGCCAGCGTGGTGGTGTCGATGG | 32 | CGA | *lacI* | 3235 | - |
| 24 | 2.3 | -2 | S30 | GTGTTCCCCGCGCCAGCGGGGATAAACCG | AGTTGGTAGCTCAGCGAACCTTGAGAAAACCA | 32 | AAG | Ori | 2140 | + |
| 24 | 2.3 | -1 | S13 | GTGTTCCCCGCGCCAGCGGGGATAAACCG | CTTGATGGTCGGAAGAGGCATAAATTCCGTCA | 32 | ATG | Kan | 1140 | + |
| 25 | 2.1 | -3 | S29 | GAGTTCCCCGCGCCAGCGGGGATAAACCG | AAcagatTTTGGTGAGTGCGGTCCTCCAACCC | 32 | AAG | Ori | 2090 | + |
| 25 | 2.1 | -2 | S8 | GAGTTCCCCGCGCCAGCGGGGATAAACCG | CTGACGACCGGGTCTCCGCAAGTGGCACTTTT | 32 | AAG | Bb | 469 | + |
| 25 | 2.1 | -1 | S34 | GAGTTCCCCGCGCCAGCGGGGATAAACCG | TTGTAATTCTCATGTTAGTCATGCCCCGCGCCC | 33 | AAG | Bb | 2319 | + |
| 25 | 2.3 | -2 | S4 | GTGTTCCCCGCGCCAGCGGGGATAAACCG | AAACCGCTGCTGCGAAATTTGAACGCCAGCAC | 32 | AAG | Bb | 210 | + |
| 25 | 2.3 | -1 | S26 | GTGTTCCCCGCGCCAGCGGGGATAAACCG | CCAGAGGTGGCGAAACCCGACAGGACTATAAA | 32 | AAG | Ori | 1723 | + |
| 26 | 2.1 | -1 | S28 | GAGTTCCCCGCGCCAGCGGGGATAAACCG | GCGCAGCGGTCGGGCTGAACGGGGGGTTCGTG | 32 | AAG | Ori | 1928 | - |
| 27 | 2.3 | -1 | S16 | GTGTTCCCCGCGCCAGCGGGGATAAACCT | GATGTTACAGATGAGATGGTCAGACTAAACTG | 32 | AAT | Kan | 1174 | - |
